# The General Practitioner and Consumption

**Published:** 1918-03-02

**Authors:** 


### The Hospital

The Workers' Newspaper of Administrative Medicine and Institutional Life, Administration, National Insurance and Health.

No. 1656, Vol. LXIII. SATURDAY, MARCH 2, 1918. PRICE ONE PENNY

### THE GENERAL PRACTITIONER AND CONSUMPTION.

The feeling in favour of the establishment of a Ministry of Health is acquiring a very wide distribution. One sees that afresh almost every week. About the latest indication of the kind occurred at a conference of representatives of the City Corporation and Metropolitan Borough Councils held at the London Guildhall with the Lord Mayor as chairman. This meeting passed a unanimous resolution that the time had come for reform of the unsatisfactory overlapping system existing with regard to the treatment of tuberculous persons in London by the formation of one central authority; this authority to be responsible for the administrative control of all matters concerning tuberculous persons, whether insured or non-insured. Well, it will have its hands full, which is precisely why a tuberculosis section of the Ministry of Health (to deal with the provinces as well as with the capital, we hope) is really a necessity. This new *ad hoc* body will have many deficiencies to supply, and tangles to unravel, and faults to set right. It need not be afraid of root and branch reform, for that is merely what is expected of it. In short, in approaching the task it will doubtless listen to the promptings of common sense and begin at the beginning.

As, for instance, by considering the responsibilities of the man who has to make the first, and therefore the most difficult, diagnosis, who has to pick out tuberculosis from common coughs and colds, from anæmia, from pneumonias, from sprains and diarrhœas and hysteria and heart disease and malingering, practically from everything else. Such is his task, in the accomplishment of which he is unfortunately heavily handicapped. First of all, in the matter of time. Before a patient has got his clothes off and been fully inspected, percussed and auscultated, and got them on again, and been talked to and advised and prescribed for, twenty minutes will have gone by. What panel doctor (and three-fourths of general practitioners are panel doctors) can spare twenty minutes for one audience only of each suspicious case of tuberculosis? And next, the medical attendant is handicapped in the matter of practice, for the most of his work is made up of trivial ailments and midwifery. Physical examination of a decisive character he gets out of the way of very soon. These handicaps in this present period of war shortage are increased to a crushing degree. If early diagnosis of consumption, which everybody—patient, expert, family doctor, relations—cries for, is to be attained, why then, taking the thing by and large, it is useless looking to the general practitioner. But who is talking platitudes now? (says the objector the conscientious writer ought to be always imagining)—this is where the tuberculosis officer comes in. Does not every prattler on tuberculosis say that the tuberculosis officer is well employed in visiting, helping, and encouraging the medical practitioners in his district? He does, and his advice is sound, were it possible; and were the tuberculosis officer always a real expert. There is a source of evidence one is loth to draw on, because those furnishing it were heroes. Still, if Voltaire's saying hold that we owe to the dead nothing but truth, one may point out that the obituary notices of tuberculosis officers perishing in the war show that a surprising proportion had no real tuberculosis experience at all before taking up their appointments.

The relations between general practitioners and tuberculosis officers we believe to be, in actual fact, as follows. Eighty per cent. of the cases coming to the dispensary are sent by, say, 5 or 10 per cent. of the doctors of the district. Taking the postulate made at the end of the last paragraph as granted, the relations between these two or three doctors and the tuberculosis officer are beneficent and heuristic and praiseworthy. Every doubtful case in their practices is sent at once for an opinion. The tuberculosis officer examines carefully, at least twice, and writes fully his diagnosis to the medical man concerned, giving in detail the physical signs, especially those easily recognisable. Disease is nipped in the bud, patients are pleased, the general practitioner tactfully educated, mutual gratitude abounds, good feeling flourishes all round. But the relations between the tuberculosis officer and the more careless large majority who only now and again send a patient for diagnosis, are not thus ideal. Some of these men, never recognising phthisis until it is well-rooted and growing fast, have a good-natured scepticism of its curability which is immensely hard to shake. Useless to point out to them instances such as that of the professional champion of a game most of them are fond of! They class consumption with inoperable cancer. It is a convenient view, too, for their own interest, because a gloomy prognosis is always a safeguard, and because it saves them trouble and exertion. Thus, in many tuberculosis dispensary areas, large sections remain strongholds of the disease. The problem thus presented is very real and actual and pressing. How to solve it time does not yet reveal. The tuberculosis officer may try persuasion, but as a new broom he has to beware of raising too much dust, and, moreover, he is usually in the position of a young man trying to get older ones to alter their ways, never a very hopeful position, especially in the case of doctors. The old saw, *Medicus medicum odit* contains a good deal of truth. Sometimes the local Insurance Committee notice for themselves the peculiar geographical incidence of dispensary patients we have endeavoured to describe, and do something to let the negligent practitioners become aware that it has been noticed. Upon this there may ensue a slight temporary improvement. The aforementioned war shortage of medical men naturally, and in many respects rightly, makes those remaining somewhat independent in their attitude towards lay control however. Nevertheless, in instances where the public health suffers, and the present is such an instance, it is hard to see how the beneficent effect of some slight measure of compulsion can be rightly avoided. This is where the prestige of a central body may be of service.

